# Roadmap on multifunctional materials for drug delivery

**DOI:** 10.1088/2515-7639/ad05e8

**Published:** 2023-12-21

**Authors:** Benjamin Nottelet, Sytze Buwalda, Cornelus F van Nostrum, Xiaofei Zhao, Chao Deng, Zhiyuan Zhong, Ernest Cheah, Darren Svirskis, Chloe Trayford, Sabine van Rijt, Cécilia Ménard-Moyon, Ravi Kumar, Nermin Seda Kehr, Natan Roberto de Barros, Ali Khademhosseini, Han-Jun Kim, Tina Vermonden

**Affiliations:** 1 IBMM, Univ Montpellier, CNRS, ENSCM, Montpellier, France; 2 Department of Pharmacy, Nîmes University Hospital, Univ Montpellier, 30900 Nimes, France; 3 MINES Paris, PSL University, Center for Materials Forming, 06904 Sophia Antipolis, France; 4 Utrecht Institute for Pharmaceutical Sciences, Utrecht, The Netherlands; 5 Biomedical Polymers Laboratory, College of Chemistry, Chemical Engineering and Materials Science, and State Key Laboratory of Radiation Medicine and Protection, Soochow University, Suzhou 215123, People’s Republic of China; 6 School of Pharmacy, University of Auckland, 85 Park Road, Grafton, Auckland 1023, New Zealand; 7 Department of Instructive Biomaterials Engineering, MERLN Institute for Technology-Inspired Regenerative Medicine, Maastricht University, PO Box 616, 6200 MD Maastricht, The Netherlands; 8 CNRS, Immunology, Immunopathology and Therapeutic Chemistry, UPR 3572, 67000 Strasbourg, France; 9 Physikalisches Institute and Center of Soft Nanoscience, University of Münster, Münster, Germany; 10 Terasaki Institute for Biomedical Innovation, Los Angeles, CA 90274, United States of America; 11 College of Pharmacy, Korea University, Sejong 30019, Republic of Korea; 12 Department of Chemistry, Izmir Institute of Technology, Izmir, Turkey

**Keywords:** polymer, hydrogels, nanomaterials, smart, drug delivery, multifunctional materials

## Abstract

This Roadmap on drug delivery aims to cover some of the most recent advances in the field of materials for drug delivery systems (DDSs) and emphasizes the role that multifunctional materials play in advancing the performance of modern DDS_s_ in the context of the most current challenges presented. The Roadmap is comprised of multiple sections, each of which introduces the status of the field, the current and future challenges faced, and a perspective of the required advances necessary for biomaterial science to tackle these challenges. It is our hope that this collective vision will contribute to the initiation of conversation and collaboration across all areas of multifunctional materials for DDSs. We stress that this article is not meant to be a fully comprehensive review but rather an up-to-date snapshot of different areas of research, with a minimal number of references that focus upon the very latest research developments.

## 1. Introduction

### Benjamin Nottelet^1,2^

^1^IBMM, Univ Montpellier, CNRS, ENSCM, Montpellier, France

^2^Department of Pharmacy, Nîmes University Hospital, Univ Montpellier, 30900 Nimes, France

### Overview

Medicine relies for a large part on active pharmaceutical ingredients (APIs) that are designed to eradicate, manage, or reverse a disease. Generally, the use of APIs alone does not result in reaching these objectives as their efficiency depends among others on pharmacokinetic aspects including absorption, distribution, metabolism and excretion [1]. To control these aspects, drug delivery is of prime importance. The concept of drug delivery encompasses numerous notions from the route of administration to the type of formulations, and the type of systems used to locate, retain or transport therapeutics in the body. Such systems are referred to as drug delivery systems (DDSs) whose main role is to ensure that API can be easily administered and delivered to the target tissue as needed to minimize side effects and optimize their therapeutic efficacy.

DDSs look back on 70 years of history with the Spansule^®^ coating technology being reported as the first example of a DDS allowing a controlled (delayed in that case) release of API and opening the way towards modern DDSs. The development of DDSs was a slow process that is classically divided in three eras [2]. The first generation, from ca. 1950–1980, focused on the acquisition of fundamental understanding on the drug release mechanisms like dissolution, diffusion, osmosis and ion-exchange. The second generation, from ca. 1980–2010, witnessed the development of the so-called smart DDSs with extensive use of polymers -especially degradable polyesters- and lipids, to develop gels and depots, micro- and nanoparticles (NPs) associating the mechanisms of the first generation with more advanced features like degradation, environmental sensitivity, targeted systems, etc. Many of these systems are the keystones of the current third generation of DDSs aiming at further expanding the release times, fine-tuning the response to physiological triggers and efficiently managing the delivery of biologics and biosimilars (antibodies, SiRNA) [3] which are now well established in the modern therapeutic arsenal beside small molecules [4].

### Open challenges

Despite a long history and countless successes both on the academic and industrial sides, the development of DDSs continues to face challenges of different nature to maintain and even expand their impact on human health.

A first challenge that remains is to achieve an efficient and robust drug loading, i.e. without premature payload release, to guarantee therapeutic outcomes with minimal adverse effects. It requires in-depth understanding of the nature of the interactions in play between API and DDSs, which is even more critical for the formulation of new generation therapeutics based on larger biomolecules, like protein and nucleic acid-based therapeutics. The effectiveness of the delivery at the right time, right dosage and right place remains also an acute matter for both small and large molecules, which requires more controlled release, more efficient targeting strategies and cross-seeding between chemistry, materials sciences, and biological sciences.

A second challenge for chemists and material scientists is to control their material at all stages, from its synthesis which must guarantee a total absence of toxicity, to its degradation and fate in the body. It requires the development of safe, bio-orthogonal chemical strategies that are inert in biological systems but allow to respond to the design complexity that is required to develop efficient DDSs. It also implies the necessity to fully understand the interactions between the components of the DDS (e.g. organic vs inorganic in hybrid systems) at different scales (e.g. macrogels with NPs). Finally, it also requires the use of labeling tools allowing to follow the DDS *in vivo* without modifying their physico-chemical properties, that are easy to implement, that are not legally constraining (e.g. radiolabeling) and that offer very high detection sensitivity.

Finally, one major challenge is the bench-to-bedside translation which raises problems of different nature. First the translation of efficient DDSs *in vitro* towards efficient DDSs *in vivo* needs more predictive *in vitro* models and models closer to *in vivo* conditions (e.g. organoïds). Second, improvements in technologies and fabrication processes to ensure controlled and reproducible synthesis, scalability, but also stability of DDSs in time before use. Last, the regulatory aspects that must be considered from the early design onwards and encompass all the above cited challenges.

Although polymers helped overcoming parts of these problems and remain predominant in the field as witnessed by the ‘pharmapolymers’ [5], none of these challenges for the future of DDSs can be met without multifunctional materials. The dynamism of the field and future breakthroughs depend on the ability of the scientific community to design, develop and transfer such multifunctional materials including non-conventional polymers for conducting [6], actuating [7] or self-healing [8] DDS, but also carbon-based DDSs [9], silica based DDSs [10], metal-organic frameworks (MOFs) [11] and hybrid systems thereof.

### Roadmap organization and aim

This Roadmap on drug delivery aims at covering some of the most recent advances in the field of materials for DDSs and especially emphasizes the key role of multi-functional materials in improving the performances of modern DDSs in relation with the above listed challenges.

The Roadmap includes different sections with a description of the current status, several current and future challenges, and a perspective of required advances in biomaterial science to tackle these challenges.

Experts in biomaterials and drug delivery have contributed to cover a broad range of topics including organic biomaterials (e.g. smart hydrogels, conducting polymers (CPs), etc), inorganic materials (e.g. silica-based systems) and hybrid or composite materials. In line with the requirements for innovation in producing DDSs, this work also addresses the concepts of microfluidics applied to multi-functional materials for DDSs.

The main objective of this document is therefore to give an overview of the current state-of-the-art of the role of multifunctional materials in the drug delivery field, to draw the attention on the main challenges that researchers could tackle to advance the area and to give directions on how to achieve it.

## 2. Smart hydrogels for drug delivery

### Tina Vermonden and Cornelus F van Nostrum

Utrecht Institute for Pharmaceutical Sciences, Utrecht, The Netherlands

### Status

Hydrogels are materials that can retain a high amount of water (typically 80%–99% water) and they are generally held together by a 3D-network of hydrophilic polymers. The most well-known and one of the early applications of hydrogels was pioneered by Lim and Wichterle with the use of hydrogels as contact lenses in the 1960’s. Since then, hydrogels consisting of a great variety of polymers have been developed and numerous biomedical applications have been reported including in the fields of tissue engineering and drug delivery.

**Figure 1.**
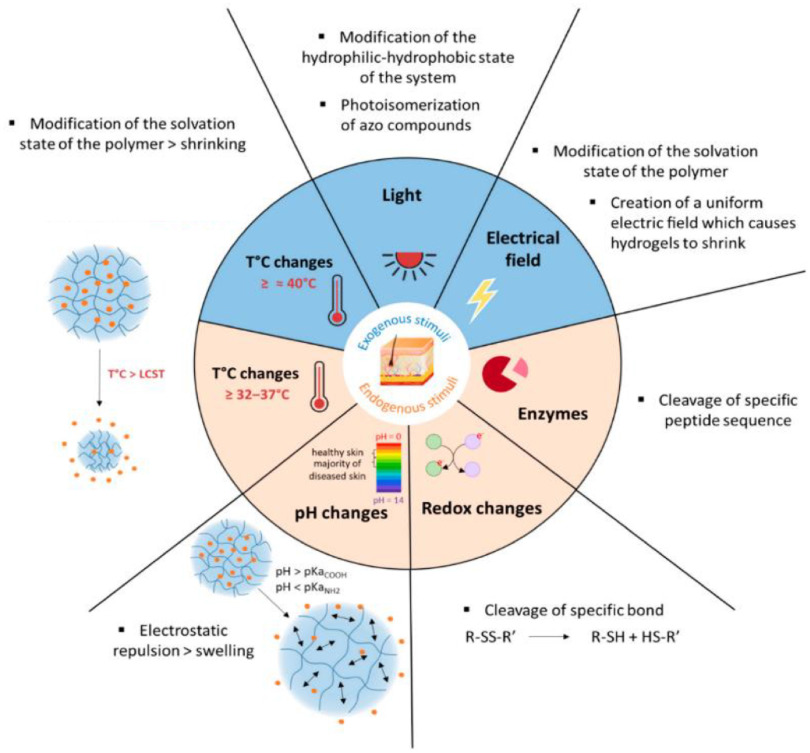
Schematic overview of stimuli and mode of action used for the design of smart drug delivery systems. Reproduced from [14]. CC BY 4.0.

The appearance of the term ‘smart materials’ originates from the developments towards materials that have the ability to respond to a certain internal or external trigger. For hydrogels that generally means that they form, swell, shrink or fall apart upon a change in pH, temperature or concentration of a certain key molecule (e.g. glucose, glutathione, enzymes or reactive oxygen species) (figure 1). More rarely used triggers to yield responsive hydrogels are based on exposure to light, ultrasound or a magnetic field. These smart or stimuli responsive hydrogels are especially of interest for delivery of therapeutic agents to a targeted location in the body. Moreover, the stimuli responsive character is also often employed to obtain injectable formulations that gel at the desired location by e.g. a change in temperature or pH.

(Smart) hydrogels are currently clinically applied or tested for a wide range of diseases at different sites in the body, for e.g. topical drug delivery and as wound dressings delivering antibacterial agents to prevent infections, ocular drug delivery and several subcutaneous and vaginal drug delivery strategies [12, 13]. So far, most clinical applications concern delivery of conventional low molecular weight drugs. However, because of the high water content of hydrogels, they are generally highly compatible with hydrophilic biological compounds and therefore of high interest for controlled delivery of the newest generation of therapeutic agents, like biologicals and cellular products, such as extracellular vesicles.

### Current and future challenges

Although several hydrogel systems have reached clinical approval, the main challenge for translational purposes is the intrinsic different behavior of hydrogels and their related drug release profile in a laboratory setting (*in vitro*) compared to an *in vivo* setting. For simple hydrogel systems, drug release profiles generally differ significantly *in vivo* from *in vitro* due to differences in degradation rates of the hydrogels. *In vitro*, physiological conditions can only be mimicked up to a certain extent, such as control over pH and temperature. In the body, besides the presence of variable concentrations of enzymes, such as proteases or polysaccharide cleaving enzymes, also mechanical forces applied to the materials play an important role in the overall stability of the hydrogel. For smart hydrogels, also the tunability of the trigger to release drugs may be changed by the presence of body fluids and external factors such as mechanical forces.

One of the areas in which hydrogels have seen enormous progress in developments is the field of ophthalmology. Starting from the initial design in contact lenses, they are now explored as injectable formulations for sustained delivery of various types of drugs including low molecular weight drugs (e.g. dexamethasone) and high molecular weight antibodies. For most ophthalmological applications, transparency of the hydrogel materials is essential to ensure undisturbed passage of light to the retina [15]. Also for applications in wound healing, a transparent material can be considered as beneficial as the healing process can be easily visualized [16]. However, transparency is often hampered in smart hydrogels in which electrostatic interactions or large hydrophobic domains are responsible for the assembly of polymers.

For smart hydrogels, both synthetic and natural building blocks are being explored. Synthetic polymers have the advantage that their properties can be fine-tuned by the design and choice of monomers. However, the synthesis of these building block often requires the use of (toxic) catalysts or coupling agents. On the other hand, natural polymers as building blocks for smart hydrogels benefit from generally milder and more biofriendly preparation methods. In any case, it is of utmost importance to obtain sterile products without any toxic or inflammatory components. Although sterilization of polymers can be done by filtration, UV or autoclaving, these methods are often not suitable for the final water containing hydrogel materials including sensitive drug substances.

### Advances in science and technology to meet challenges

Several new generation therapeutics, like protein and nucleic acid-based therapeutics need to be delivered inside the target cells. These therapeutics need to be protected from premature degradation during their travel in the body, and they need to overcome several barriers [17]. Conventional bulk hydrogels are able to protect the therapeutics, but in order to transport them inside cells, the DDSs need to be designed at a much smaller dimension, namely at the nanoscale. The intracellular space is known to offer a reductive environment due to elevated glutathione concentrations compared to the extracellular matrix (ECM). Smart reduction-sensitive nanogels make use of this trigger to either release their cargo or disintegrate such that the cargo is set free only intracellularly [18].

Since hydrogels generally have a very open structure, preventing burst release of low molecular weight cargo is a major challenge. Several options have been proposed to tackle premature burst release, including encapsulating cargo into NPs before entrapment into the hydrogel, or making use of (multi)layered hydrogels or polymer-coatings [19, 20].

Moreover, considering the interest in delivery of fragile biomacromolecules or other biological cargo such as extracellular vesicles requires that the chemistry needed to prepare the hydrogels is compatible with these sensitive molecules. Crosslinking mechanisms need to be carefully chosen to prevent unwanted side reactions with the encapsulated biotherapeutics, but also with components present in the body when in situ forming hydrogels are used. Current trends are therefore focusing on bio-orthogonal crosslinking methods that make use of functional groups that are inert in biological systems [21].

Smart nanogels can also be triggered to release drugs from a matrix or membrane as shown by the elegant examples of Hoare *et al* and Lin *et al* [22, 23]. Such a membrane consisted of encapsulated magnetic particles and thermosensitive nanogels, in which the nanogels fill up most of the space, limiting drug release from the membrane compartment. Upon a magnetic trigger, the system heats up, resulting in shrinking of the nanogels leaving open space for the drug to freely diffuse and rapidly release. The thermosensitive trigger is a smart way to induce hydrogel shrinking, which opens several opportunities for the design of multifunctional materials for drug delivery in general.

### Concluding remarks

Smart hydrogels have seen an amazing increase in attention over the past few decades and numerous triggers for drug delivery are currently being investigated. Although certain challenges still remain to be solved for widespread biomedical applications, these steps are gaining attention and therefore smart hydrogels including smart nanogels are expected to have a large impact for the effective delivery of both conventional low molecular weight drugs and novel biotherapeutic agents.

## Acknowledgments

The Dutch Research Council (NWO/VICI 18673) is acknowledged for funding (T Vermonden).

## 3. Polypeptide for drug delivery

### Xiaofei Zhao, Chao Deng and Zhiyuan Zhong

Biomedical Polymers Laboratory, College of Chemistry, Chemical Engineering and Materials Science, and State Key Laboratory of Radiation Medicine and Protection, Soochow University, Suzhou 215123, People’s Republic of China

### Status

Similar to natural proteins, synthetic polypeptides possess fascinating features (versatile functionalities, tunable architectures, unique secondary structures, excellent safety, etc), and have received overwhelming attention from biological, medical and pharmaceutical fields [24]. In the past decades, various polypeptides, glycopolypeptides, and their hybrids have been developed through recombination synthesis, solid-phase peptide synthesis, and ring-opening polymerization [25]. With decent biodegradability, biocompatibility, and facile introduction of functional groups, polypeptides are regarding as superb materials for the construction of various DDSs. Polypeptide-drug conjugates as a straightforward subtype of delivery systems were initially developed in the last century through one-step formation of amide or ester bonds between drugs and pending carboxyl/amine groups of poly(L-glutamic acid) (PGlu), poly(L-lysine) (PLys), and poly(L-aspartic acid) (PAsp) [26]. Particularly, Vivagel^®^ and Copaxone^®^ have been employed to treat immune diseases (human immunodeficiency virus, multiple sclerosis) in clinic, and several conjugates (CT-2103, CT-2106, DEP^®^DOCETAXEL, etc) have entered clinical evaluation. The strategy usually provides slow and sustained release of conjugated elements. To facilitate the precise control of drug release at lesion sites, bioresponsive linkers including hydrazone, ketal, and disulfide bonds have been introduced into the polypeptide-drug conjugates. Cabral *et al* pioneered the polypeptide-based micelles that are capable of physical encapsulation of different drugs through hydrophobic interactions for cancer treatment [27]. Particularly, a phase III clinical trial of NK-105 developed from 4-phenyl-1-butanol-modified poly(ethylene glycol)-*b*-PAsp (PEG-*b*-PAsp) and paclitaxel (PTX) have demonstrated comparable antitumor efficacy and less adverse events compared to Taxol^®^ in patients with metastatic or recurrent breast cancer [28]. Meanwhile, polypeptide-based micellar formulations of doxorubicin (DOX), SN38, and oxaliplatin have reached clinical trials to treat primary and metastatic tumors. Later, polypeptide-based polymersomes have been exploited to achieve encapsulation of hydrophobic drugs in the membrane, and hydrophilic drugs, proteins, and nucleic acids in the watery cores [25]. Considering that most of the developed DDSs are based on PGlu, PLys, and PAsp, the introduction of other amino acids (21 amino acids are used for protein synthesis in human), targeting moieties, and responsive linkers would enrich the functionalities and facilitate the construction of more sophisticated and advanced DDSs.

### Current and future challenges

Polypeptide-based drug conjugates and micellar drugs have demonstrated reduced side effects compared with free drugs in the clinical trials, however, their therapeutic outcomes are modest and far from the expectation. Thus, some core challenges need be resolved to optimize polypeptide-based DDSs. First, the driving force (e.g. hydrophobic interactions) between drugs and polypeptide moieties in micelles and vesicles are relatively weak, often causing drug escape prior to reaching lesion sites that largely compromises the therapeutic outcomes. Thus, efficient and robust drug loading in delivery systems is prerequisite to guarantee the pharmaceutical effect and bearable adverse effects. Second, recent studies showed that the average dose of NPs reached solid tumors following *in vivo* administration was less than 1% [29]. Thus, strategies used to overcome physiological barriers, elevate tumor deposition, and cellular internalization of nanomedicines would be critical to improve therapeutic efficacy. Folate, peptide, polysaccharide, aptamer, antibody, and antibody fragments have been reported to be effective ligands to elevate drug enrichment at target sites [30]. Third, conventional nanomedicines often afford slow drug release upon arriving at target sites [31] that might hinder the therapeutic effects and cause drug resistance. Fourth, many clinical studies verified that single therapeutics usually brought about unsatisfied outcomes, due to the molecular complexity and heterogeneity of most diseases.

### Advances in science and technology to meet challenges

**Figure 2.**
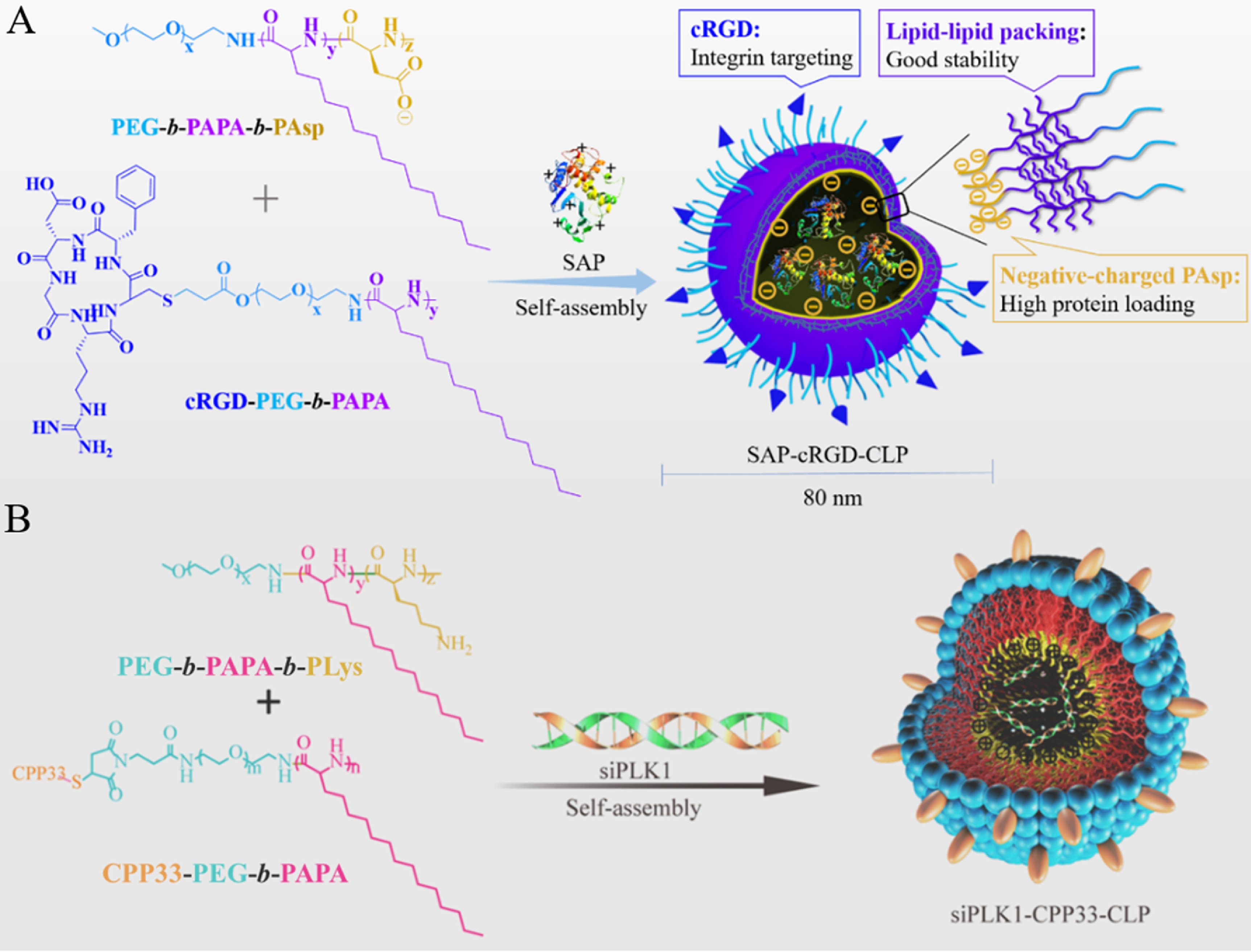
Schematic illustration on preparation of chimaeric polymersomes from PEG-*b*-PAPA-*b*-PAsp (A) and PEG-*b*-PAPA-*b*-PLys (B) copolymers for quantitative loading of proteins and siRNA, respectively. Reprinted with permission from [33]. Copyright (2018) American Chemical Society. [34] John Wiley & Sons. © 2019 WILEY-VCH Verlag GmbH & Co. KGaA, Weinheim.

In micellar formulations, the hydrophobic interactions between drugs and polypeptide moieties are typically utilized for drug encapsulation, while often result in modest drug loading capacity and poor *in vivo* stability. Recently, various physical interactions including *π-π* stacking, lipid–lipid packing, hydrogen-bond, donor-acceptor, and bundled helical structure have been exploited to achieve robust and efficient drug loading [32]. For example, micelles formed from hydrophobic poly(*β*-benzyl-L-aspartate) and poly(L-tyrosine) demonstrated up to 63.1 wt% DOX loading content via *π–π* stacking, and micelles from poly(*α*-aminopalmitic acid) (PAPA) afforded decent encapsulation of docetaxel (DTX) and peptide drugs. Polymersomes as superior alternatives to liposomes gain great attention for the delivery of both chemical agents and biopharmaceutics [25]. Especially, chimaeric polypeptide-based polymersomes consisting of PEG shell, hydrophobic polypeptide membrane, and charged hydrophilic core have been recently exploited from asymmetric triblock copolypeptides. The chimaeric polymersomes based on PEG-*b*-PAPA-*b*-PAsp and PEG-*b*-PAPA-*b*-PLys afforded almost quantitative loading of proteins and siRNA via electrostatic interactions (figure 2) [33, 34]. To meet the requirement of stable during circulation while destabilized at lesion sites, dynamically stabilized polypeptide-based vehicles have been recently pursued by introducing responsive (pH, redox, photo, etc) linkers between drugs and polypeptides, reversibly covalent crosslinking of vehicles, and strong physical interaction among vehicles [31].

Cellular uptake plays a critical role for the nanomedicines that need reach the intracellular target. Studies signified that the incorporation of targeting ligands (antibody, antibody fragments, aptamer, peptide, polysaccharide, etc) into polypeptide-based DDSs greatly boosted their cellular uptake, subcellular organelle attack, and penetration of physiological barriers like blood-brain barrier [30]. Besides, Song *et al* have developed α-helical polypeptides pending with cationic side-chains that demonstrated superior cell-membrane permeability over conventional cell penetrating peptides (e.g. TAT, Arg9), and up to 9-fold higher transfection efficiency of DNA than commercial 25 kDa branched polyethylenimine *in vivo* [35]. The molecular complexity and heterogeneity of most diseases requires the combination of different therapies including chemotherapy, radiotherapy, and immunotherapy. Especially, immune-related agents that can activate antigen presenting cells, recruit immune cells, inhibit immune checkpoint, and reprogram immunosuppressive cells emerge as imperative elements to combine with chemical agents and radiotherapy to optimize the therapeutic efficacy.

### Concluding remarks

In the past decades, polypeptide-based nanomedicines with robust drug loading, efficient cellular uptake, decent drug deposition and controlled drug release at target sites have been exploited and demonstrated exciting therapeutic progress. However, several issues need be improved to accelerate their clinical translation: (i) synthetic techniques need be improved to gain large-scale polypeptides with defined molecular weights and amino acid order at low cost; (ii) although the unique features of polypeptides like secondary structure, functional groups have been utilized to modulate the drug delivery processes (drug loading, physiological barriers penetration, tissue distribution, etc), however, it is still in a preliminary stage, need be further explored; (iii) sophisticated design of polypeptide-based vehicles is required to attain co-encapsulation of different agents include chemical drugs, radioactive isotope, immune-related agents, proteins, and nucleic acids (mRNA, siRNA, miRNA, etc) to combat the complexity and heterogeneity of most diseases.

## Acknowledgments

The financial support from the National Natural Science Foundation of China (NSFC 51973149, 51773145, and 51633005) and the Natural Science Foundation of the Jiangsu Higher Education Institutions of China (19KJA220002) is acknowledged.

## 4. Conducting polymers for drug delivery

### Ernest Cheah and Darren Svirskis

School of Pharmacy, University of Auckland, 85 Park Road, Grafton, Auckland 1023, New Zealand

### Status

In 1977, Shirakawa *et al* found that by doping polyacetylene, the organic polymer could be altered to exhibit metal-like conductivity [36]. This discovery spawned the development of an exciting group of electroactive materials, collectively termed CP. CPs have since found applications in a wide range of electronic, bioelectronic, and biomedical fields, including for drug delivery.

The redox state of CPs can be modified by chemical or electrical stimuli which alters the charge, volume, and permeability of the material; this can be exploited to achieve stimuli-responsive drug delivery. Initial experiments using CPs for drug delivery in the 1990s focused primarily on the delivery of charged drug molecules such as dopamine and salicylate. In these early experiments, several inherent limitations of CPs were highlighted [37]. Primarily, CPs faced difficulties in the incorporation of a wide range of drug payloads, requiring small charged drug molecules to electrostatically interact with the CP. Furthermore, only low levels of drug loading could be achieved, and inconsistent release profiles were reported.

**Figure 3.**
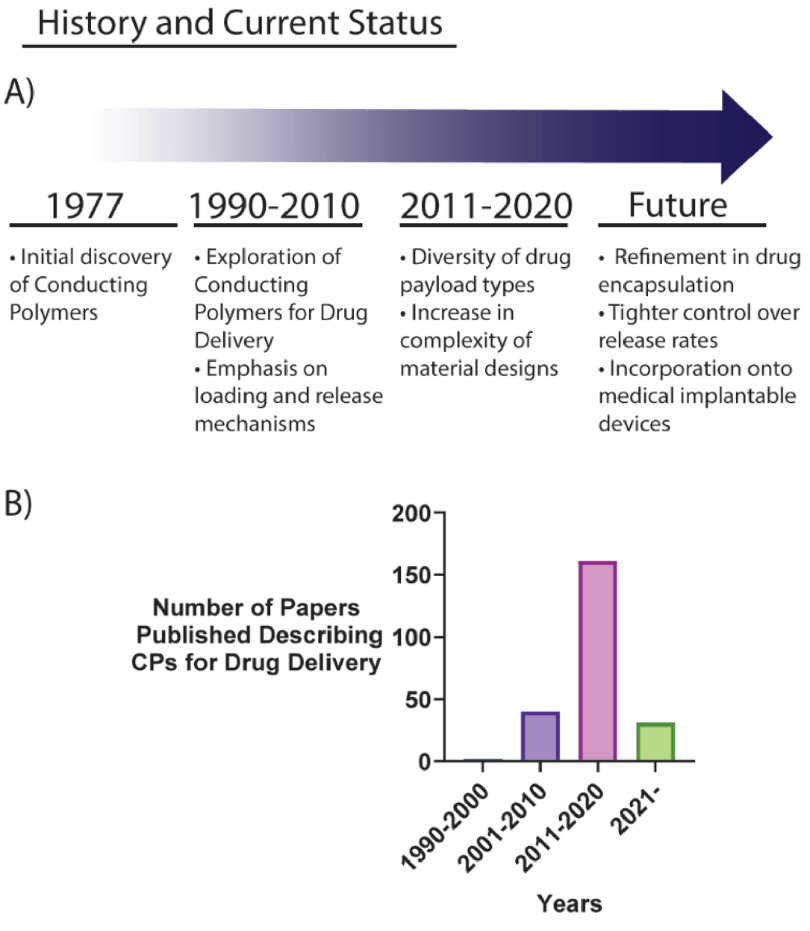
(A) Key historical highlights of the CP drug delivery field. (B) Number of papers published within the CP drug delivery field indexed by SCOPUS.

In the last two decades, the formulation of more complex designs of CPs has been explored to overcome these limitations. Various nanostructures and composite materials, primarily with hydrogels, have been reported to improve the drug delivery capabilities of CPs [38, 39]. Subsequently, the range of drug payload types have increased, extending towards neutral compounds such as progesterone, and even large macromolecules (figure 3).

The CP innovation field is still in its infancy, with no commercial DDS currently available. However, CPs remain highly attractive due to the promise of electrically triggered delivery systems. The ability to alter release rates of a therapeutic according to the changing microenvironment of the body as a disease or injury progresses is alluring, particularly in the context of personalized medicine.

Future advancements within this field will see increased refinement in drug encapsulation methods and tighter control over release rates. On-demand drug delivery capabilities could be added onto medical implantable devices, increasing their utility. These advancements will no doubt yield viable uses of CPs in the medical landscape in the future.

### Current and future challenges

**Figure 4.**
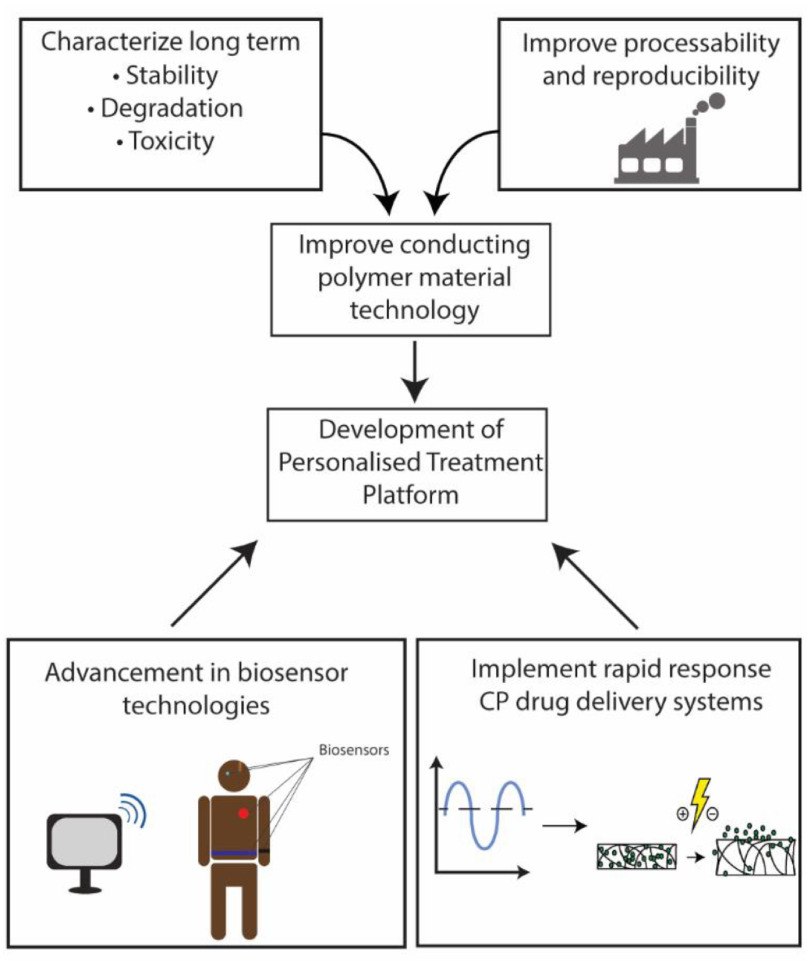
Summary of the challenges facing the field of CP drug delivery systems and the advancements needed to achieve translation into the clinic.

Research into CPs for drug delivery has focused on increasing the versatility of drugs that can be delivered, increasing drug loading capacity and control over release rates. Mechanistic investigations remain important in addressing these challenges to further develop these materials as efficient DDSs. Challenges to further advance CP DDSs towards the clinic can be grouped into two broad categories: (1) improvements in technologies and fabrication processes surrounding CP materials and (2) co-evolution and integration of the CP drug delivery technology with bioelectronic platforms to fit clinical requirements (figure 4).

Further maturation of CP-related technologies is required for clinical adoption. As part of the preclinical safety and regulatory data needed for timely translation into an approved delivery system available to patients, researchers should be cognizant of characterizing the stability, degradation, and potential toxicity from CP based delivery platforms. Other challenges in this area include improving dosage dispensation control and preventing unintended drug escape. In addition, CP DDSs have been historically developed in small scale and in controlled laboratory conditions. Ultimately, to achieve the future goal of commercialization, scalable processes need to be developed and demonstrated, specifically improving the processability and reproducibility of the material using green chemistry approaches wherever possible. Improving these manufacturing processes to enable efficient scale up represents a significant challenge.

The plethora of wearable biosensors and modern neurotechnology devices such as the cardiac, cochlea, and deep brain implants already add benefit to the lives of millions of people today. In conjunction with these devices, CP DDSs offer an opportunity to afford a highly targeted and localized drug dosing, effectively extending their capabilities of bioelectronic devices. Utilization of biological information from these devices to inform the dosage requirement represents the pinnacle of personalized medicine. CP delivery systems should be developed concurrently with a clinical treatment platform in mind, with the end goal of developing a system capable of monitoring relevant biomarkers and subsequently generating an appropriate treatment in real time. Building such a platform represents a major challenge and would require a multidisciplinary team effort.

### Advances in science and technology to meet challenges

Fundamental understanding of biology and material sciences has made tremendous advances within the field and will continue to do so into the future. To address the challenge of maturing CPs, we look towards the development of functionalization of the CP itself and the use of composite materials to enhance stability and performance of CP DDSs. For example, composite materials containing a CP and a biocompatible hydrogel can increase drug loading capacity while ensuring a biocompatible electrode-tissue interface [39]. Meanwhile, precise and selective drug delivery can be achieved using next generation electrophoretic flow devices. These devices display highly targeted drug release control enabling the delivery of high-speed local neurotransmitters pulses [40].

Typical CP fabrication approaches are compatible with existing photolithography techniques. To assist in scale up in the fabrication of CPs, innovations and techniques from the electronic manufacturing sector can be adapted and applied. Micro- or even nano-level microfabrication techniques are currently being developed at scale [41]. Therefore, it stands to reason CP DDS can benefit greatly from adopting these techniques.

The incorporation of a CP delivery system on a treatment platform is a significant challenge, however, its trajectory remains promising. Inroads to accomplish this goal require the advancement and integration of both biosensor technologies and rapid response CP DDSs. As an example of biosensor technology advancement, Vomero *et al* described a highly stable, conformable electrocorticography microelectrode array capable of recording multi-unit activity of the brain [42]. In a closed loop system, this data can then be utilized with a rapid response DDS. Muller *et al* described such a system through the rapid release of an anti-epileptic drug from a CP after a detectable seizure event [43]. While the system developed in this work used pre-recorded seizure data and the drug delivery remained *in vitro*, the system provides a powerful proof of concept as a strategy towards a fully integrated, autonomous implantable system.

Technologies like these, alongside advancements in AI technology enable coupling of CP DDSs with sensors. Drug delivery is thereby controlled by a feedback loop, a format which could be applied to a wide range of treatment platforms.

### Concluding remarks

CP drug delivery technologies hold great promise within the medical devices industry. The ability to alter drug delivery rates on-demand provides incentive to pursue development. However, further research to optimize both the methods and fabrication techniques are still required for this technology to reach its full potential. Additionally, this technology will likely progress in parallel with, and in response to, advances in other areas such as implant and biosensor fields. Due to the tight integration with these fields, multidisciplinary teams are paramount for its success. Rapid advancements in the last 5 years have positioned the field of CP drug delivery for translation to the clinic with commercial viability. With a multidisciplinary approach, CP drug delivery technology will be utilized as a successful platform for medical treatments.

## Acknowledgments

E C acknowledges support from the Vernon Tews Educational Trust. D S acknowledges support from a Sir Charles Hercus Health Research Fellowship (19/007) from the Health Research Council of New Zealand.

## 5. Silica based systems

### Chloe Trayford and Sabine van Rijt

Department of Instructive Biomaterials Engineering, MERLN Institute for Technology-Inspired Regenerative Medicine, Maastricht University, PO Box 616, 6200 MD Maastricht, The Netherlands

### Status

Mesoporous silica NPs (MSNs) are NPs typically in the range of 20–200 nm with an ordered porous structure, and are popular materials in the field of drug delivery [44]. Their popularity stems from their large surface area and pore volume, which allows efficient biomolecule loading of various cargo including hydrophilic and hydrophobic drugs. Moreover, their tunable structure and possibility for surface and core functionalization’s make MSNs incredibly flexible platforms to design multifunctional nanomaterials not only for drug delivery, but also for catalysis, sensing and bioimaging applications [45].

**Figure 5.**
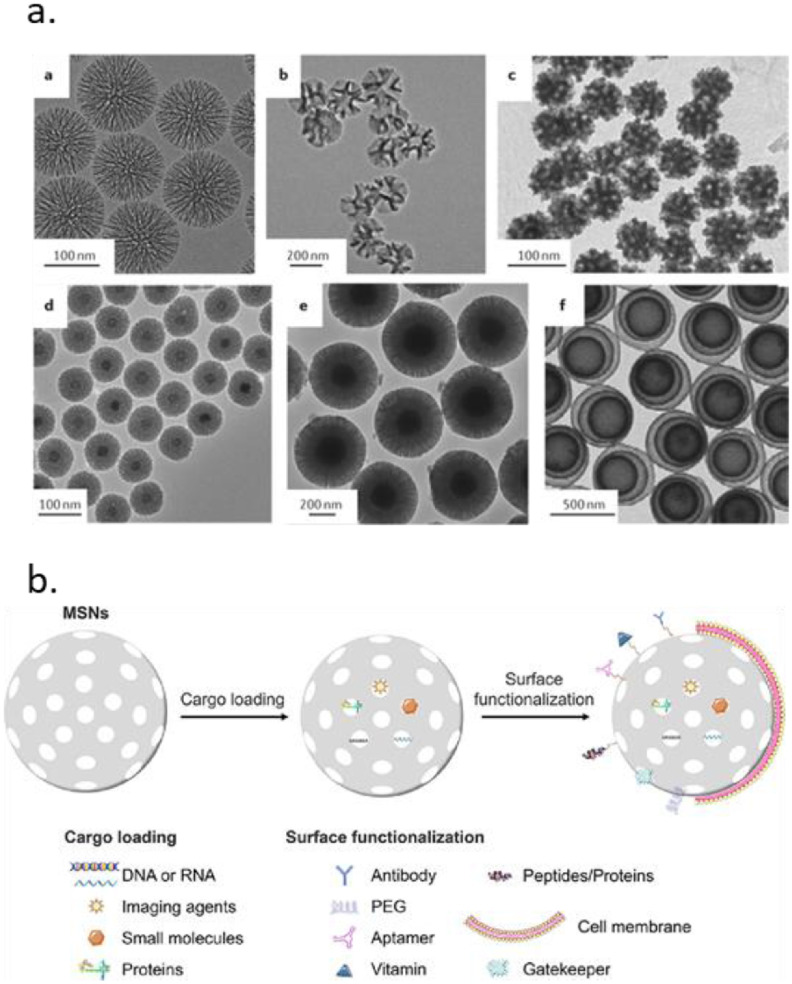
(a) Electron microscopy images of MSNs with different architectures. Reproduced from [47], with permission from Springer Nature. (b) Examples of MSN cargo loading and surface functionalization strategies. Reprinted from [48], Copyright (2020), with permission from Elsevier.

MSNs are synthesized through hydrolysis and condensation of silica precursors in the presence of a surfactant acting as a structure-directing agent and were first introduced as drug carriers in 2001 [46]. Over the past decades, MSNs with various morphologies, sizes, and pore structures have been developed (figure 5(a)) [47]. A major advantage of MSNs is the possibility for selective surface modifications to create carrier systems with tissue targeting and controlled drug delivery features (figure 5(b)) [48]. There are especially many examples of gating systems that allow on demand cargo delivery using internal (e.g. pH, enzyme levels) or external (e.g. light, magnetism) stimuli. In these strategies, the MSN surface is modified with stimuli sensitive linkers to which gating molecules are tethered. Additionally, the silica surface can be modified with lipid bilayers, or functionalized with cell or tissue targeting moieties to allow efficient intracellular delivery of cargo in specific tissues. Moreover, the matrix of MSNs may be doped or core modified with imaging agents such as fluorescent probes or metal NPs to enable bio-imaging [45]. As a result, a plethora of options exist for creating multifunctional tailor made MSNs for specific applications.

As such, MSNs represent promising tools to overcome constraints associated with the use of conventional drugs. These include low solubility, low stability, and lack of specificity. MSNs can improve the pharmacokinetics of therapeutic agents by allowing the incorporation of large amounts of cargo within their core structure, providing protection from degradation. This combined with the possibility for on demand cargo release and tissue targeting can drastically improve the effectiveness of the incorporated therapeutics while lowering possible side-effects.

### Current and future challenges

The largest challenge for MSNs, like that of many nanomedicines, is bench-to-bedside translation. In the past decades preclinical studies mainly done in small rodents have shown that MSNs are tolerated at relatively high doses, and do not cause a thrombotic response [49]. A phase 1 clinical evaluation of silica NPs for PET imaging of solid tumors were deemed safe and ready for further clinical trials [50]. Although highly encouraging for the field, these silica particles are ultra-small (∼6 nm) and surface modified with PEG polymers. It is well known however, that shape, size and surface chemistry strongly impact how NPs are processed *in vivo* [51] and thus for each MSN formulation a new safety assessment needs to be performed. Another challenge in their clinical evaluation is large-scale synthesis including reproducibility under good manufacturing practices (GMP). This becomes more challenging with more sophisticated particle designs.

By far most of the reported literature on MSNs focuses on their use in the cancer research field with articles running in their many thousands. Although the use of MSNs in other clinical applications such as for vaccines, creams, diagnostics, imaging or in the regenerative medicine field is also demonstrated the available knowledge on more sophisticated drug delivery strategies has not been fully utilized in these fields [52]. Implementing such strategies will provide the community with (more) sophisticated tools to allow tighter control over biological processes, important for many therapeutic strategies.

Although many parameters of MSN synthesis have been optimized, using MSNs for the delivery of larger biomolecules is complicated by the size restriction of the pore size, which typically ranges from 2–10 nm. Meanwhile the use of RNA, DNA and proteins in therapies is surging but is hindered by the lack of an efficient cell delivery system. MSNs have high potential as genetic delivery system due to their large loading capacity, extensive functionalization possibilities, and on demand release properties. In the optimization of MSNs towards large biomolecule encapsulation two main approaches have been used including enlarging the pore size and creating a hollow core [53]. However, the efficiency of biomolecule loading, and targeted release remains a significant roadblock.

### Advances in science and technology to meet challenges

Despite the rapid development of multifunctional MSNs, knowledge of the *in vivo* biological effects of these nanomaterials is lacking. The effect of size, in-vivo degradation, and impact of surface chemistry on pharmacokinetics and biocompatibility are critically important parameters that need to be investigated to be able to assess their safety. Also, different therapeutic strategies require different administration routes which influence the pharmacokinetic and biocompatibility characteristics of MSNs and need to be investigated separately (e.g. for use in creams or vaccines). To assist these studies, methods should be optimized for *in vivo* tracing of MSNs with simultaneous monitoring of their behavior including stimuli-responsiveness, target specificity, and degradation. Further, industry needs to play a role by developing reliable methods for large-scale MSN synthesis with batch-to-batch reproducibility under GMP conditions. This reproducibility is important for the standardization of drug loading levels but also includes the optimization of more elaborate MSN designs such as with metal or fluorescent doping, enlarged pores and hollow cores. Once different types of MSNs can be manufactured reproducibly at large-scale, their transition to preclinical screening and subsequent human clinical trials is made wholly easier.

To develop MSNs as reliable tools also for large biomolecule delivery, advances are needed to improve the loading and targeted release efficiency. MSNs should be capable of active loading of biomolecules and subsequent targeted, time-controlled release. This may be achievable by the synthesis of MSNs that conjugate to or electrostatically complex with biomolecule cargo and further degrade in intracellular environments. The translation of MSNs as cost-effective multifunctional materials for the delivery of various biomolecules in other research fields is already under way, with successful demonstrations in biocatalysis, regenerative medicine and diabetes.

### Concluding remarks

In the past decades there has been a lot of progress towards the development of MSNs for stimuli-responsive, controlled and targeted delivery of drugs, especially in the cancer therapy field. Additional chemical modifications can extend their use to allow the delivery of larger biomolecules such as genetic material and proteins. Moreover, proper implementation of knowledge and tailoring of MSNs will ensure their widespread use in many scientific fields.

To facilitate their clinical translation, improved understanding on how MSNs behave in complex *in vivo* settings is needed. While reliable and large-scale synthesis of MSNs is a prerequisite to clinical screening. Regardless of these challenges, it is clear that MSNs bring significant benefits as cost-effective multifunctional materials for the delivery of various biomolecules and are expected to continue to have a large impact on the drug delivery field.

## Acknowledgments

We would like to thank LINK limburg (S van Rijt) and ZonMW (Chloe Trayford) for funding.

## 6. Carbon-based nanomaterials as carriers for drug delivery

### Cécilia Ménard-Moyon

CNRS, Immunology, Immunopathology and Therapeutic Chemistry, UPR 3572, 67000 Strasbourg, France

### Status

The biomedical applications of carbon-based nanomaterials represent a field in continuous expansion [54, 55]. These materials include 0D fullerenes, graphene quantum dots (GQDs) and carbon dots, 1D carbon nanotubes (CNTs), carbon nanohorns and graphene nanoribbons, 2D graphene family nanomaterials and 3D nanodiamonds. This section focuses on the most widely used nanomaterials as carriers for drug delivery, namely CNTs, graphene (and its oxidized form, graphene oxide), carbon dots and GQDs. Graphene is an atomically thin honeycomb lattice consisting of sp^2^-hybridized carbon atoms. A CNT can be represented as a graphene sheet rolled up to form a cylinder. Carbon dots are quasi-spherical NPs (amorphous, potentially with sp^2^ carbon clusters), while GQDs have an atomically thin graphitic plane (generally 1 or 2 layers) with a circular or elliptical shape. Both dots have a size typically less than 10 nm and possess abundant oxygenated groups that endow them with good water dispersibility.

Carbon nanomaterials have the capacity to adsorb a variety of drugs on their surface through *π*–stacking and/or electrostatic interactions. Their high specific surface area gives the possibility to load one, or more different drugs, providing a potential synergy to avoid drug resistance and the incidence of relapses. Thanks to their capability to absorb near-infrared light and convert it into heat, the drug release can be photothermally controlled. Numerous combination therapies can also be investigated, such as chemo and photothermal therapy, photodynamic therapy and chemotherapy, or gene and chemotherapy.

Although carbon nanomaterials share similar properties, they also have distinct features. Compared with CNTs, graphene has a higher specific surface area, enabling to reach higher drug loadings. The cavity of the CNTs can be exploited to load drug molecules, and both single- and multi-walled CNTs can be excited in the near-infrared region (700–1100 nm) by two-photon fluorescence microscopy. Owing to their small size, carbon dots and GQDs allow developing compact nanocarriers for delivering drugs to specific regions inaccessible for graphene and graphene oxide. They display remarkable optical properties that depend on their size, surface chemistry and shape, making them useful for bioimaging and theranostics [56]. Moreover, GQDs can cross the blood-brain barrier, offering opportunities for the treatment of central nervous system diseases. They also have an activity against multidrug resistance, as they can downregulate associated transporter genes.

Even if carbon nanomaterials are predominantly in preclinical testing, they have great potential for biomedical applications. However, some challenges need to be addressed before a future clinical translation.

### Current and future challenges

There are some concerns about the use of carbon nanomaterials in nanomedicine, including biosafety issues, a low biodegradability, a poor aqueous solubility (for CNTs and graphene), which can be circumvented by chemical functionalization, and a less well-defined structure compared to other types of nanomaterials.

**Figure 6.**
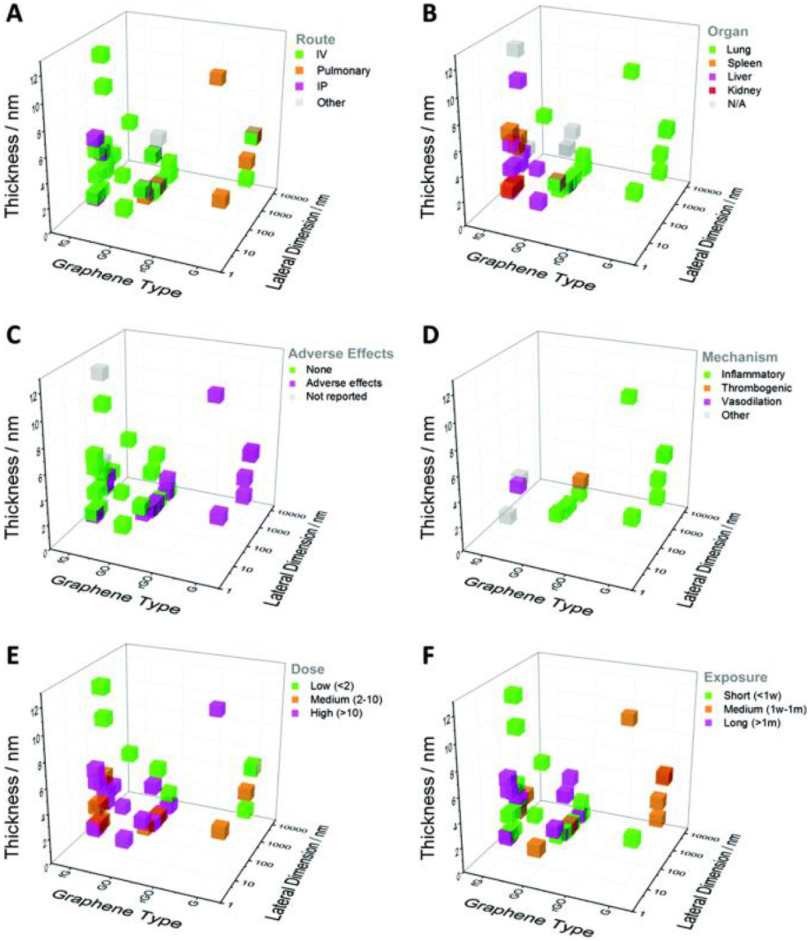
Impact of the structure (thickness, lateral size) and composition of graphene family nanomaterials on their *in vivo* safety. G: graphene, rGO: reduced graphene oxide, GO: graphene oxide, fG: functionalized graphene. (A) Impact of the mode of administration, IV: intravenous, IP: intraperitoneal. (B) Organs where the graphene family nanomaterials accumulate the most. (C) Reported reported adverse effects or not. (D) Mechanism responsible for the adverse effects. (E) Impact of the administered dose, low dose: <2 mg kg^−1^, medium dose: 2–10 mg kg^−1^, high dose: >10 mg kg^−1^. (F) Impact of the exposure duration, short: exposure <1 week, medium: between 1 week and 1 month, long: >1 month. Each reported graphene family nanomaterial is represented by a cube and is positioned in the graph according to the type of graphene, its average thickness and lateral dimension. Reproduced from [57] with permission from the Royal Society of Chemistry.

The toxicity of carbon nanomaterials is the foremost issue to address. Many studies have pointed out the distinct effect of some physicochemical features, such as the size, shape, surface properties, and purity, on their pharmacokinetics and safety, allowing to alleviate toxicity by chemical functionalization (figure 6) [57]. Compared with graphene and CNTs, GQDs and carbon dots have an improved biocompatibility, which is mainly related to their small size and high water dispersibility. However, their use in nanomedicine is still in their infancy. Overall, it is difficult to generalize the potential toxicity or biosafety of carbon nanomaterials. More detailed toxicity studies have to be performed in other models than mice and rats, such as zebrafish, aquatic frogs and primates, in particular to assess potential long-term adverse effects. In addition, in agreement with the principles of the 3Rs (Replacement, Reduction and Refinement), the development of organoids and organ-on-chips, which mimic complex physiological conditions and recapitulate human organ function, helps minimizing animal experiments and alleviate ethical issues related to animal studies by means of more predictive and representative preclinical systems. In this context, more studies should be performed to assess the potential toxicity of carbon-based nanomaterials using organoids and organ-on-chip systems. Besides, some toxicity data are controversial; this might be due to the variability and inhomogeneity between samples. For example, although the functionalization of CNTs enhances their biocompatibility by increasing their water dispersibility, some nanotubes can aggregate, resulting in potential health risk. This aggregation can be explained by the asymmetric distribution of hydrophilic groups on their surface.

As carbon nanomaterials were initially presumed to be biopersistent, it is fundamental to assess their biodegradability. They can be degraded by peroxidases (e.g. myeloperoxidase) secreted by immune cells, such as neutrophils, eosinophils, and macrophages [58]. Nevertheless, long-term degradation studies (over a period of several months) are limited and require further investigation to fully assess the safety of carbon nanomaterials after *in vivo* administration.

Another critical challenge is related to a lack of controlled synthesis leading to well-defined and homogeneous carbon nanomaterials. Indeed, CNTs have variable diameter, number of walls and length. Graphene family nanomaterials can vary in terms of the lateral size, thickness (although graphene is monolayer *per se*) and oxidation state in the case of graphene oxide. In the literature, GQDs and carbon dots are often not carefully distinguished. The lack of standardization in the production of carbon nanomaterials hampers their translation into clinical trials.

### Advances in science and technology to meet challenges

The toxicity effects of carbon nanomaterials can be alleviated or avoided by chemical functionalization. For example, the asbestos-like pathogenicity of long and pristine multi-walled CNTs was totally reduced when they were functionalized with tri(ethylene glycol) chains bearing ammonium groups, with a concomitant decrease of their length, resulting in the disaggregation of the nanotubes [59].

**Figure 7.**
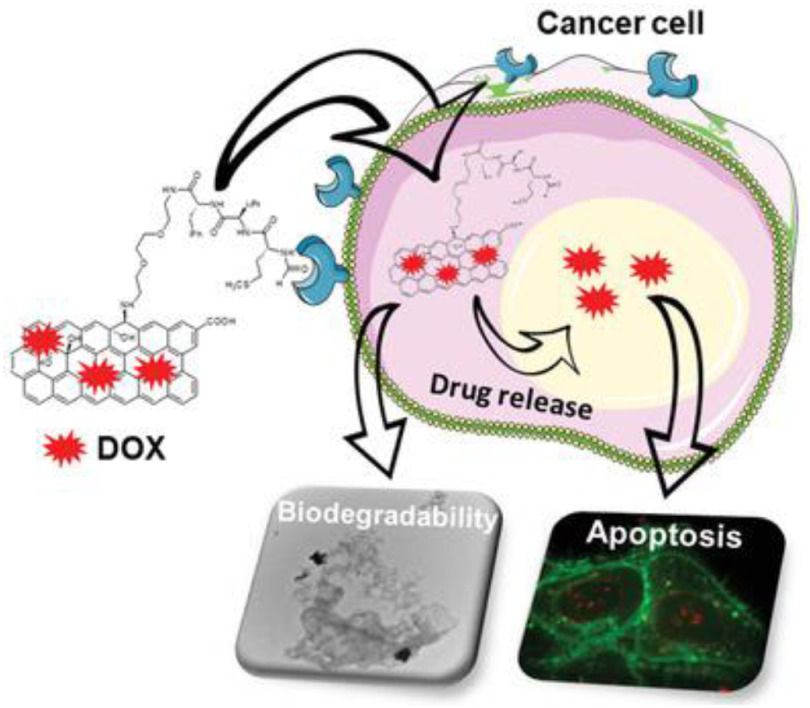
Multifunctional graphene oxide covalently conjugated to a peptide able to target cancer cells and enhance the biodegradability of the material by myeloperoxidase, and with doxorubicin adsorbed on its surface, resulting in cell apoptosis. [60] John Wiley & Sons. © 2019 WILEY-VCH Verlag GmbH & Co. KGaA, Weinheim.

The biodegradation of carbon nanomaterials allows avoiding bioaccumulation, thus minimizing their long-term toxicity, as long as the toxicity degradation products are not harmful. Degradation-by-design strategies rely on the functionalization of the nanomaterials with molecules able to enhance their biodegradability. Such approaches include their derivatization with substrates of enzymes to attract them in close proximity to the nanomaterials, molecules that prevent the inactivation of the enzymes and enhance their activity, or molecules with pro-oxidant effects, for instance for H_2_O_2_ generation. Developing ligands with tumor targeting properties and the capacity to enhance the nanomaterial biodegradability is of utmost importance. In this regard, graphene oxide was covalently functionalized with the chemotactic peptide *N*-formyl-methionyl-leucylphenylalanine that interacts with the formyl peptide receptor, which is expressed on the membrane of different cancer cells (figure 7) [60]. This peptide has also the capability to activate neutrophils causing the release of myeloperoxidase. The peptide-graphene oxide conjugate was used as a carrier for the targeted delivery of the anticancer drug DOX and underwent a faster myeloperoxidase-mediated degradation.

The lack of standard synthetic methods to prepare carbon nanomaterials with a well-defined structure and composition is a key challenge. The synthesis of uniform single-walled CNTs has received great attention since many years, but it has still not been achieved on a large-scale [61]. Recently, an active research effort has been put in the chemical synthesis of well-defined graphene molecules (nanographenes) by bottom-up approaches [62], but they have not been investigated for biomedical applications yet. By contrast, the use of graphene nanoribbons is a highly emerging research field in nanomedicine [63]. Graphene nanoribbons are structurally well-defined; they are narrow (<50 nm) lengthened strips of single-layer graphene. They are synthesized by covalent coupling of suitable precursor molecules and their full biomedical potential is not yet entirely exploited.

### Concluding remarks

Carbon nanomaterials are promising for biomedical applications, especially as carriers for drug delivery. Research involving CNTs in nanomedicine is an active field since more than 20 years. In addition to graphene, novel forms of carbon have recently joined the family of carbon nanomaterials, namely carbon dots and GQDs, which have very encouraging biomedical potential. To overcome the limitations associated to the structural inhomogeneity of batches produced using different methods, an emphasis should be given on the production of well-defined carbon nanomaterials, in particular graphene nanoribbons, and more efforts towards the synthesis of nanographene and uniform CNTs, carbon dots and GQDs. Chemical functionalization is crucial to minimize their potential toxicity and enhance their enzymatic biodegradability. Nevertheless, more studies are needed to assess their long-term toxicity and fate before any future clinical translation.

## Acknowledgments

The author gratefully acknowledges the support of the Centre National de la Recherche Scientifique (CNRS), in particular through the 80|Prime program, and the support of ARTHRITIS R&D.

## 7. Inorganic/organic hybrids in drug delivery

### Ravi Kumar^1^ and Nermin Seda Kehr^1,2^

^1^Physikalisches Institute and Center of Soft Nanoscience, University of Münster, Münster, Germany

^2^Department of Chemistry, Izmir Institute of Technology, Izmir, Turkey

### Status

Inorganic/organic hybrids are used for numerous applications due to their favorable physicochemical properties resulting from the combination of different inorganic and organic building blocks. In recent decades, efforts have been made to deliver drugs by encapsulating them in inorganic/organic hybrids [64–66]. These hybrids not only exhibit their individual properties, but also offer novel properties such as stimulus sensitivity, specific targeting, and controlled delivery of hydrophilic and hydrophobic biomolecules due to the synergistic combination of their organic and inorganic moieties.

Inorganic NPs offer nanoscale binding sites, large surface area, tunable pore sizes, size-dependent unique optical, electrical and mechanical properties, and thermal and chemical stability. The organic chemistry on the inorganic NPs improves biocompatibility, reduces toxicity, provides specific targeting properties, and encapsulates hydrophilic and hydrophobic biomolecules. The hybrids improve the availability and sensitivity of drug release at the required sites with controlled or specific rates. Cost-effective synthesis, controllable shape and pore size, modification by surface chemistry, inertness and stability have made them an important tool for drug delivery. Researchers have used various inorganic particles such as MSNs, periodic mesoporous organosilicas, layered double hydroxides, calcium phosphate NPs (CP NPs), metal NPs (e.g. gold NPs (Au NPs), gold nanorods (Au NRs), gold nanoshels (Au NSs), gold nanoclusters (Au NCs), silver NPs (Ag NPs)), metal oxide NPs (e.g. iron oxide (Fe_3_O_4_), carbon-based NPs such as graphene, graphene oxide (GO), fullerene (C60) and CNTs for drug delivery [64–69]. The burst release of drugs and toxicity of NPs limit the use of NPs in clinical trials. Therefore, inorganic/organic hybrids are prepared by coating the inorganic NPs with natural or synthetic polymers (e.g. chitosan, alginate, polyethylene glycol (PEG), polyethylenimine (PEI), polylysine (PLL)), cell membranes, dentrimers, and lipids to improve stability under biological conditions, provide sustained and stimulatory release, and reduce the toxic effects of high-dose drugs in the body [67]. There are also other strategies, e.g. preparation of dendrimer-based NPs, inorganic NPs encapsulated in liposomes, incorporation of organic units during the synthesis of inorganic NPs and embedding NPs in hydrogels or decorating NPs with bioactive molecules, e.g. proteins, peptides, vitamins, and carbohydrates through covalent and non-covalent interactions used for site-specific tissue binding and controlled drug delivery [64, 66, 69].

### Current and future challenges

The hybrid systems have great advantages in drug delivery for biomedical applications, but there are problems with existing hybrid systems such as undesirable interactions with serum proteins, lack of specific targeting and prolonged and slow drug delivery, toxicity due to released ions from inorganic NPs, low degradability of inorganic NPs, fast degradation of organic moieties, limited control over the desired concentration of drug delivery at the target site, and low encapsulation efficiency. Other technical challenges include reproducibility in the synthesis and functions, limited characterization techniques, and scale-up methods of the inorganic/organic hybrids. Future challenges, therefore, are to fine-tune the hybrid inorganic/organic NPs for effective therapeutic action and dosing and to understand the controlled release of drugs at specific sites at different pH levels in different parts of the body. In addition, many researchers have successfully demonstrated the potential of hybrids *in vitro*, but there are not enough studies *in vivo* to understand the fate of hybrids in clinical applications. For clinical application, it is very important to reduce the toxicity, aggregation and clearance of drugs without affecting the other part of the body and to achieve patient specific drug delivery. Another challenge is to develop multifunctional nanostructure hybrids that can deliver multiple drugs simultaneously with controlled rate/amount and increase the amount of encapsulated drugs [68].

### Advances in science and technology to meet challenges

Adaptive intelligent DDSs that operate independently are expected to address current challenges. However, such systems require significant advances in the functionality and reliability of current systems. Researchers have developed ‘smart’ DDSs in which biomolecules are introduced through self-assembly or interact with the surfaces of NPs through non-covalent interactions. Such hybrids enable stimulus-dependent drug delivery that can also regulate the released dose of drugs according to disease states in the body that produce conditions of low pH, elevated temperature, and different glucose concentrations. Currently, smart injectable hybrid systems are being developed in which NPs are incorporated into shear-thinning hydrogels to deliver the optimal concentration of drugs to the target site only when needed, limiting the nonspecific distribution of drug molecules in healthy tissues or elsewhere in the body [70]. These systems are also designed to deliver multiple drugs to a specific site, with the ability to control the delivery time and amount of each drug.

**Figure 8.**
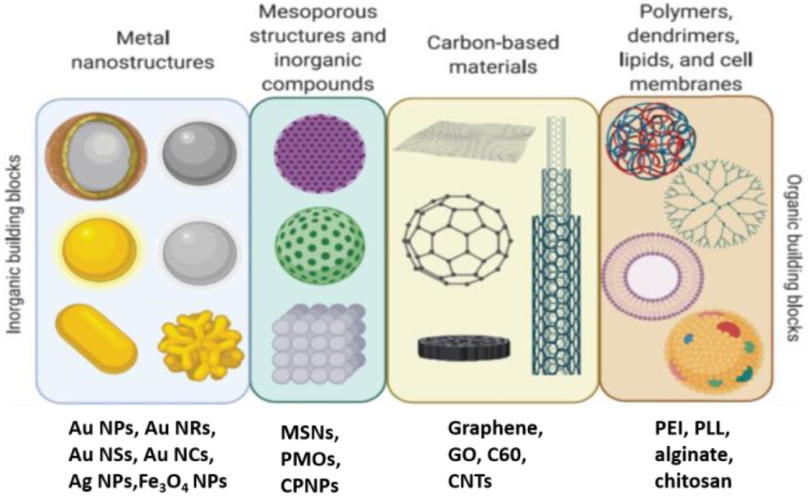
Building blocks for hybrid nanosystems. Reprinted with permission from [67]. Copyright (2021) American Chemical Society.

To efficiently use the hybrids *in vivo*, the surface of the hybrids can be modified with lipids, proteins, and cell membranes derived from specific cells (figure 8). NPs functionalized with natural cell membrane fragments are used to reduce the nonspecific interaction of the NPs with other cells, proteins, and tissues to improve site-specific drug delivery [64]. These systems can extend the circulation time of NPs in the body and improve NP biocompatibility by reducing free metal ion release, particle–particle aggregation, blood clotting, and immunotoxicity. NPs functionalized with natural cell membrane fragments of e.g. neutrophils, platelets and cancer cells are used to reduce the nonspecific interaction of the NPs with other cells, proteins, and tissues to improve site-specific drug delivery [64, 70]. These systems can extend the circulation time of NPs in the body and improve NP biocompatibility by reducing free metal ion release, particle-particle aggregation, blood clotting, and immunotoxicity. However, they cannot be used for patient-specific drug delivery because cell membrane components differ only slightly among patients. In this regard, efforts should focus on automated implantable hybrid systems that can realize patient-specific release patterns and enable optimal drug dosing and release as needed.

Further *in vivo* and clinical studies are needed to fully understand the advantages and disadvantages of these hybrids to gain complete control over the properties of inorganic-organic hybrids. Moreover, the coupling of multiple nanomaterials/organic polymers to the hybrids is expected to exhibit better therapeutic/targeted/controlled stimulus-dependent drug release efficacy than their individual counterparts.

### Concluding remarks

The combination of organic and inorganic components in hybrid systems enables systemic tuning of their properties and makes the hybrid systems potential candidates for drug delivery. Hybrid inorganic/organic NPs show promise in overcoming the current challenges of existing inorganic and organic materials in controlled, sustained, and targeted drug delivery. However, care must be taken to avoid short- or long-term adverse effects caused either by the elements released during degradation or by the non-biodegradable nature of the inorganic NPs and the uncontrolled distribution of the delivered drugs to other sites in the body. In addition, these hybrids need to be tested in preclinical tests on animal models to gain a comprehensive and deep understanding of their functions with biological components of the human body. Overall, future developments of inorganic/organic hybrids should consider the rational design of adaptive injectable hybrid DDSs with diverse [71, 72] and novel functionalities that are easily adjustable and controllable to enable fully controlled, efficient, and site-specific drug delivery.

## Acknowledgments

We thank Deutsche Forschungsgemeinschaft (KE 1577/7-1) for funding.

## 8. Nanoparticle-hydrogel composites

### Sytze J Buwalda

MINES Paris, PSL University, Center for Materials Forming, 06904 Sophia Antipolis, France

### Status

**Figure 9.**
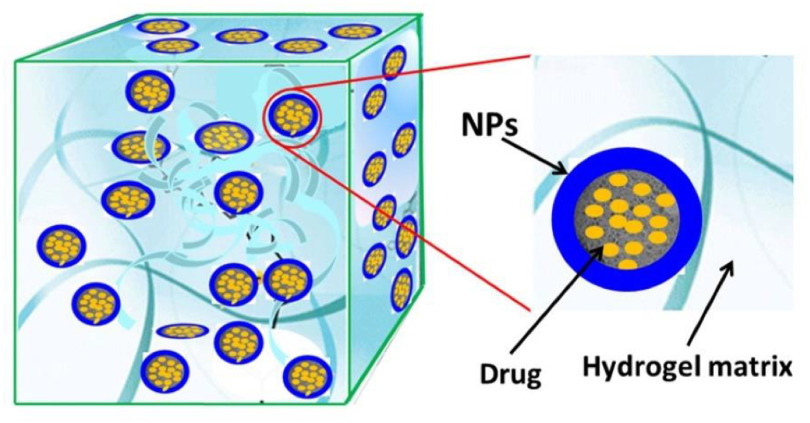
Schematic representation of a nanoparticle-hydrogel composite. Reproduced from [85]. CC BY 4.0.

Hydrogels are polymer networks that are able to retain a large amount of water in their swollen state. Following their discovery in the 1960s by Wichterle and Lim, they were initially applied as contact lenses. Later, hydrogels have been used extensively for the controlled delivery of therapeutic agents [73]. In parallel, various NP systems have been developed for drug delivery applications, including nanomaterials based on polymers (e.g. poly(lactide-co-glycolide)), carbon (e.g. CNTs), other inorganic materials (e.g. silica) and metals (e.g. gold) [74]. NPs have been used as diagnostic tool and as carriers to enhance the solubility, bioavailability, targeting and controlled release of various drugs. In the 1990s, researchers started to combine NPs and hydrogels to create composite materials (figure 9) with unique, improved and synergistic properties. Whereas early investigations focused on improving the mechanical properties of hydrogels, in the 2000s NP-hydrogel composites (NHCs) for drug delivery started to emerge. One of the first examples was reported by Lee and Chen who investigated the release of several model drugs from an acrylic acid-poly(ethylene glycol) methyl ether acrylate hydrogel containing negatively charged silica NPs [75]. It was found that the release rate from the composite system can be controlled via the interactions between the ionic charge of the drug and the negative surface charge of the NP. For example, electrostatic attractions are present between the cationic model drug crystal violet and the NP-containing matrix, leading to a relatively slow drug release. In contrast, charge repulsion exists between the anionic model drug phenol red and the matrix, resulting in a faster release. In the past two decades, the physical or covalent incorporation of NPs in bulk hydrogels has shown advantageous for drug delivery in many ways. For example, NHCs facilitate the simultaneous, controllable release of multiple drugs with varying solubility, which has important benefits over monotherapy, such as a lower risk of drug resistance and reduced side effects [76]. Furthermore NHCs may overcome disadvantages of the individual components, such as fast, uncontrolled drug release. However, as the field is still in its infancy, processing-structure-property relations are only starting to be established, making it challenging to design NHCs with optimal properties for a desired application. In the following section, several issues are discussed that should be overcome to fully exploit the potential of NHCs and facilitate their clinical application.

### Current and future challenges

Many studies on NHCs include cytotoxicity experiments, usually demonstrating the absence of toxicity to a certain cell line. However, very little is known about the effectiveness, toxicity, biodegradation and ultimate fate of these systems *in vivo*, substantiating the need for systematic studies in this direction. This is especially important since many nanomaterials have been reported to possess adverse health effects [77].

A second challenge is the poor stability of certain NP formulations in hydrogel precursor solutions, leading to aggregation and inhomogeneous distribution in the composite. Aggregation occurs mostly with hydrophobic NPs and may lead to decreased mechanical properties of the NHC, a reduced efficiency or a failure to exhibit the anticipated synergistic characteristics.

Thirdly, most NHCs are formed *ex vivo* and require surgery to be implanted and exert their function. *In situ* forming composites can be introduced *in vivo* in a minimally invasive manner (e.g. via a syringe) and provide clear advantages, including the absence of the need for surgery as well as a good fit with the surrounding tissue [76]. The challenge with such injectable systems is to combine fast gelation kinetics with a biocompatible crosslink method.

Fourthly, in most NHCs each entity exerts only a single function. For example, often the hydrogel only serves as a depot for drug-containing NPs. To better address the complex nature of tissue and to increase therapeutic efficacy, multifunctional NHCs are highly sought after.

Fifthly, there is a need to clarify the interactions between the NPs and the polymer network of hydrogels. Especially the release mechanism of embedded NPs is an important item, as a controlled release of NP-incorporated drug from the composite is often needed and excessive NP leakage may cause toxicity.

Lastly, stimulus-responsiveness of composite systems has received only limited attention. A number of NHCs have been designed to respond to a single trigger, such as a change in pH. However, multistimulus-responsive NHCs, which allow for enhanced control over the responsive behavior, are still very scarce.

### Advances in science and technology to meet challenges

The aggregation of NPs in composites may be prevented by introducing repulsive interaction forces, which can be achieved by increasing the surface charge (electrostatic stabilization), by preventing NPs from approaching at close distances (steric stabilization) or by a combination of the two mechanisms (electrosteric stabilization) [78]. The surface charge of NPs can be increased by grafting of ligands (containing e.g. a quaternary amine group) or adsorption of charged ions. Steric stabilization may be achieved via grafting of e.g. PEG chains, which generate a hydrated cloud with a large excluded volume.

Injectable composites may be realized by making use of bioorthogonal crosslinking techniques, which do not interfere with biological processes upon covalent crosslinking. Such reactions are also known as chemoselective processes or, more colloquially, as click chemistry. This expression was introduced in 2001 by Kolb *et al* [79] for several regiospecific linking reactions giving high yields and requiring little to no purification, of which the Cu(I)-catalyzed Huisgen 1,3-dipolar cycloaddition of terminal alkynes and azides is the best-known example. Since then, a number of other bioorthogonal reactions have been employed for the crosslinking of polymers into hydrogels [80], such as Staudinger ligation, thiol-ene and thiol-yne chemistry as well as classical and inverse electron demand Diels-Alder cycloaddition. Furthermore, strain-promoted azide-alkyne cycloaddition between cyclooctyne and azide groups has been shown to result in fast, efficient and biocompatible crosslinking without the need for an external stimulus or catalyst.

Investigation of the *in vivo* performance of NHCs may be facilitated by advances in non-invasive imaging techniques, which enable spatiotemporal monitoring of nanosized biomaterials and allow for a reduction in the number of animals. NPs are either intrinsically detectable (e.g. via the recently developed Magnetic Particle Imaging technique) or they can be functionalized with imaging probes via various conjugation techniques. In order to visualize the release of NPs from the composite and their subsequent biodistribution, multimodality imaging techniques in combination with multimodal NPs (e.g. fluorescently labelled iron oxide particles which can be detected via fluorescence imaging and magnetic resonance imaging) are preferred as they can provide more information compared to a single imaging modality by combining advantages and compensating for limitations [81].

Multistimulus-responsiveness may be achieved by preparing multicomponent networks such as (semi)-interpenetrating networks. This strategy has been used for the preparation of dual responsive composite systems at the micro- and nanoscale with little interference between the two stimuli (e.g. silver NPs in poly(acrylic acid)-poly(N-isopropylacrylamide) microgels, displaying temperature and pH sensitivity [82]) but could potentially also be extended to macroscopic NHCs.

Clarifying the interactions between NPs and hydrogels may be achieved via a combination of spectroscopic techniques. For example, Zewde *et al* used Fourier transform infrared spectroscopy to investigate interactions in a composite consisting of albumin NPs and a hyaluronic acid-poly(ethylene glycol) hydrogel [83]. The stretching and out of plane bending O–H peak of the hydrogel was shown to have broadened in the composite in comparison with the neat hydrogel, suggesting hydrogen bonding in the composite. In the same study, x-ray photoelectron spectroscopy data suggested that the hydrogel network rearranges in the composite material so that poly(ethylene glycol) is pointing up toward the surface while hyaluronic acid folds to interact with the albumin of the NPs. Also rheology may be used to determine the mechanisms behind the enhancements afforded by the incorporation of NPs. For example, Zaragoza and Asuri performed rotational rheological characterizations of chemically crosslinked poly(acrylamide) hydrogels with embedded silica NPs [84]. They found that pseudo-crosslinking, governed by noncovalent interactions between the NPs and the hydrogel polymers, plays an important role in the observed reinforcements.

### Concluding remarks

Research over the past two decades has clearly demonstrated the interest of incorporating NPs in hydrogels for drug delivery applications as the composites combine the beneficial properties and overcome the limitations of each component. This contribution highlighted six major challenges the research area currently faces, which relate to NP aggregation, the need for *in situ* formation, the lack of knowledge regarding *in vivo* performance and NP-hydrogel interactions, as well as a need for multifunctionality and multistimulus-responsiveness. Several advances in science and technology, including recent chemical, spectroscopic and non-invasive imaging techniques, were proposed to address these challenges. These advances may assist researchers in the rational design of NHCs which can perform multiple tasks via ingenious combinations of different functional capabilities. Research in the proposed directions may lead to the development of the next generation of NHCs and may facilitate their translation into clinically feasible, multifunctional drug delivery solutions.

## 9. Multifunctional materials for microfluidics related to drug delivery

### Natan Roberto de Barros^1^, Ali Khademhosseini^1^ and Han-Jun Kim^1,2^

^1^Terasaki Institute for Biomedical Innovation, Los Angeles, CA 90274, United States of America

^2^College of Pharmacy, Korea University, Sejong 30019, Republic of Korea

### Status

**Figure 10.**
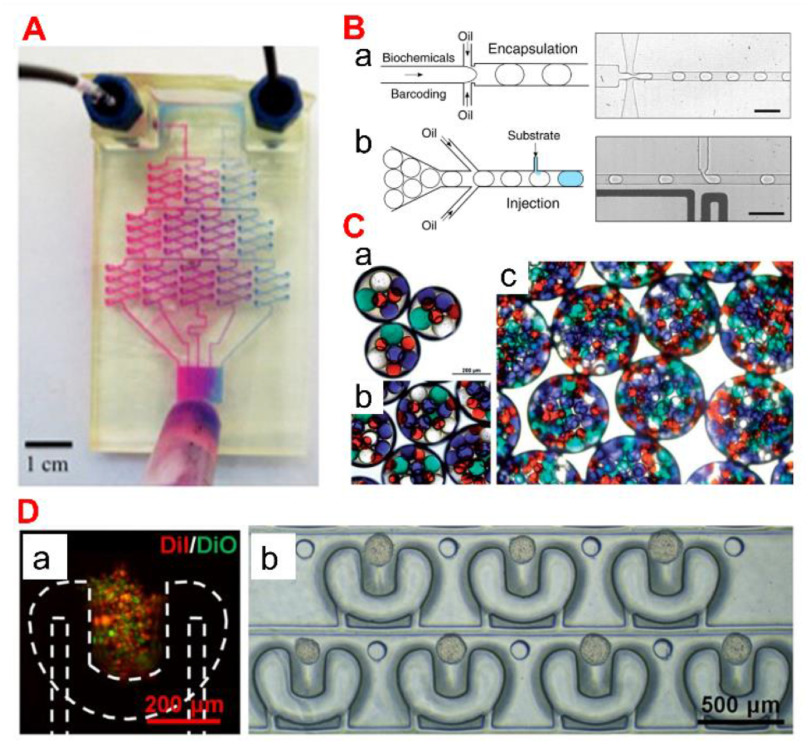
(A) A microfluidic gradient generator with three levels of combining, mixing, and splitting, resulting in different drug ratios. Reprinted with permission from [92]. Copyright (2014) American Chemical Society. (B) A microfluidic platform where biochemical components are encapsulated in droplets stabilized by a surfactant. Reproduced from [93]. CC BY 4.0. (C) Double emulsions with four different types of inner drops: (a), (b) small numbers of inner drops and (c) high density of inner drops. Reproduced from [94] with permission from the Royal Society of Chemistry. (D) A microfluidic platform for cell localization and 3D tumour production. (a) Cell trapping. (b) 3D tumor array cultured for 2 d. Reprinted with permission from [95]. Copyright (2019) American Chemical Society.

Drug delivery is the method or process of administering therapeutics [86]. Oral, inhalation, or subcutaneous injection are the most common methods of drug administration. However, due to the body’s physical and biological barriers, the treatment is potentially ineffective. Microfluidics, a sophisticated technology applied to pharmaceutical nanotechnology, has revolutionized a wide range of therapeutic delivery strategies. Microfluidics is the science of designing and engineering fluid flows at the microscale. This allows the processing of very small volumes of fluids in microchannels to establish advanced DDSs with high precision and sensitivity (figure 10(A)). For instance, microfluidic platforms can be engineered to perform drug synthesis, local and on-demand or continuous/sustained drug delivery, and micro- and NP production (figures 10(B) and (C)). In particular, the development of multifunctional materials applicable to this microfluidic platform ranges from long-term treatment through sustained release from the DDS to responsive treatment that responds to the environment of the delivery site, and smart treatment that responds to externally controllable stimuli. In microfluidics-based drug delivery strategies, many different types of materials have been studied, and multifunctional materials that can be used as drug carriers include (1) polymers [87], (2) peptides [88], (3) lipids [89], and (4) inorganic materials (ceramics [90], metals [91]). For the development of these functionalized drug carriers, not only the inherent properties of the material such as drug carrying capacity, solubility, stability, and reactivity, but also the biological properties related to the target disease such as ease of clinical use, toxicity and biocompatibility, and bioactivity (prevention/treatment) are considered.

Another promising possibility among microfluidics-based approaches in the pharmaceutical sector is drug toxicity and efficacy testing. Drug development is an important area of medical research with one of the most challenging and costly investments by governments worldwide. Despite these huge investments, many drugs fail to reach their goals in the preclinical/clinical research stage, so innovation in drug delivery that can simplify and reduce these steps is essential. Based on these unmet needs, microfluidics-based technology has proven its role as an organ-on-a-chip (OoC) platform that can mimic the fluid flow of human tissues and ultimately mimic the microenvironment of human organs and diseases (figure 10(D)). In particular, the multifunctional material not only affects the improvement of cell viability, but also simulates the ECM surrounding the cells, enabling more sophisticated biological response analysis. Through these advances in material science and microfluidics, health and disease-based human OoC platforms promise to replace animals as preclinical models for drug development, provide more translation, and reduce the time and cost associated with current preclinical practices.

### Current and future challenges

Despite advances in materials science and microfluidics to develop advanced DDSs, translation for clinical applications remains challenging. The most fundamental problem is that there may be scalability limitations due to low yield due to high precision based on microfluidic chips. Although the pharmaceutical industry requires production of at least kilograms per day, current production rates for drug-loaded drug carriers via microfluidic platforms are on the milligram scale. In addition, depending on the design of the microfluidic chip and the nature of the drug delivery scaffold material combination, the potential for clogging and occlusion of the microfluidic chip has the potential to hinder this scalability improvement. Another possible issue is the stability of the drug carrier during manufacturing. A polymer that can be used as a drug delivery carrier through a microfluidic chip must be able to flow in the ‘microfluidic chip’ and have solid stability after particle generation. These properties are not only influenced by the material’s inherent physical properties (topography [96], elasticity [97], shear thinning properties [98]), but also by biochemical properties (similarity to ECM molecules [99], presence of fibrogenic cytokines or growth factors [100]) that can directly or indirectly affect the scaffold. Modulation of these various factors affects the stability of the drug delivery scaffold, which in turn has the potential to be optimized as a regulator of drug release.

If a microfluidics-based drug delivery scaffold is fabricated using a material that can be stabilized through a cross-linking process, an energy stimulus such as UV during the fabrication process can provide stability suitable for microfluidics-based drug delivery technologies. However, sterilization processes, particularly energy-induced terminal sterilization approaches such as steam sterilization and *γ*-irradiation, can cause denaturation, destabilization, and degradation of scaffold materials. In addition, membrane filtration sterilization, another sterilization option, is a sterilization method that is possible only when the size of particles made of microfluidic chips is smaller than the pores of the membrane. Another challenge is how to safely store and deliver drug-loaded particles generated through microfluidic devices under various physicochemical environmental factors such as temperature and humidity. The drug carrier made through the microfluidic device can be changed into a powder form that is easy to store through freeze-drying after the crosslinking process. However, there is also the possibility that multifunctional polymers or specific classes of drugs (e.g. protein) may lose their reactivity and effectiveness when the temperature changes rapidly. Therefore, it is required to develop a cryoprotectant that can maintain the shape and structure of particles with highly uniform and precise characteristics for a long period of time. A final challenge to be addressed is how to deliver particles made with microfluidic devices. For particulates loaded with drugs with high precision, the optimal drug administration route may be different from that of existing drugs depending on the functionality of the drug carrier. Additional studies on drug delivery routes and drug distribution specific to DDSs will be needed.

### Advances in science and technology to meet challenges

As mentioned above, high-throughput manufacturing of drug-loaded particles is essential for clinical translation. Research to improve scalability is being actively conducted. As part of these studies, the results show that parallelizing microchannels within a microfluidic platform can speed up the fabrication of drug carriers [101]. On the other hand, a system capable of increasing the particle production rate up to 3 kg per day has been described in recent literature [102]. The system is a coaxial turbulent jet mixer that allows for scalability of DDSs, wherein the drug delivery carriers produced through this system display the same properties as those produced at bench scale.

**Figure 11.**
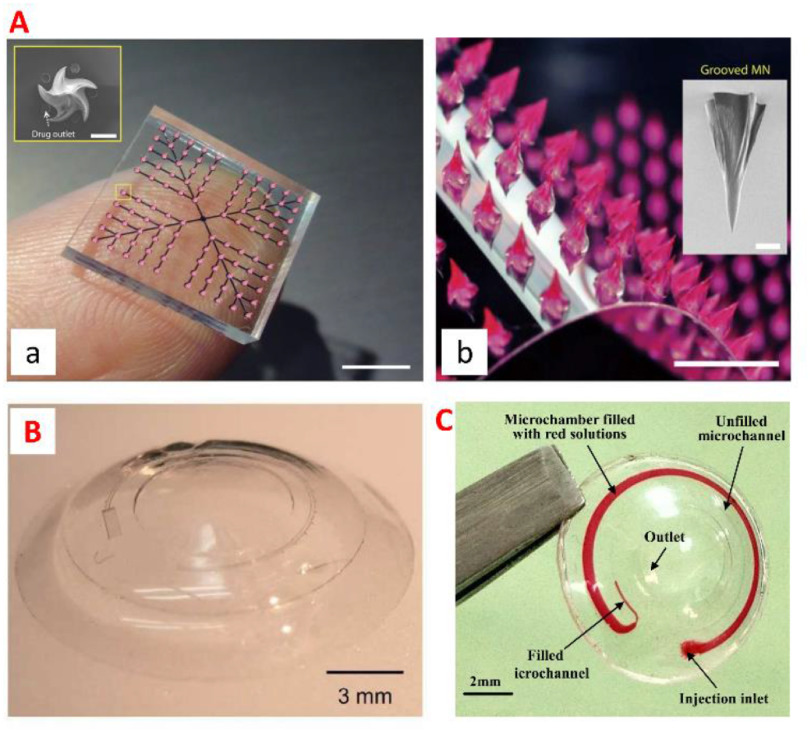
(A) A microfluidic snake fang-inspired MN array for drug delivery. The integrated MN patch consists of an MN array and a PDMS chamber that has microholes through which liquid formulations can trickle out of the chamber. From [103]. Reprinted with permission from AAAS. (B) A microfluidic strain sensor embedded contact lens. Reproduced from [104] with permission from the Royal Society of Chemistry. (C) A wearable microfluidic contact lenses with potential in the ophthalmology healthcare field. Reproduced from [105]. © IOP Publishing Ltd All rights reserved.

In the technique of delivering particulates, the microneedle-based technology may be a promising delivery strategy (figure 11(A)). The microneedle penetrates the stratum corneum of the skin with little or no pain, enabling drug delivery to be localized to the dermis layer, thereby increasing patient compliance. In addition, microfluidic integrated hollow microneedles can deliver drug formulations through multifunctional/smart materials that exhibit different release profiles depending on the design of the microfluidic platform. Furthermore, this system can be combined with a polymer capable of absorbing and releasing water in response to stimulation, enabling minimally invasive sampling and sensing applications.

The on-demand drug delivery through microfluidic platforms can be achieved by integrating biosensors within the microchannels. Eye diseases, for example, require the correct amount of the drug because an overdose can cause side effects and an underdose is ineffective. In this regard, microfluidic embedded contact lenses have been developed for drug delivery (figures 11(B) and (C)). However, in order to enable drug delivery using a microfluidic device, it is necessary to develop a coating material that can improve the mechanical mismatch between the contact lens and the eye for the biocompatibility improvement. In addition, it will be possible to develop smart contact lenses through the incorporation of smart materials that can release drugs in response to sensors and stimuli.

### Concluding remarks

The combination of material science, microfluidics, and drugs to treat/manage disease holds great promise for accelerating translation into advanced DDSs, OoC platforms, and clinical practice. However, with respect to this highly interdisciplinary field of research, microfluidic-based drug delivery platforms are still in the development stage. Challenges remain to improve translation potential by improving scalability and yield, to maintain stability in pharmaceutical processes, and to achieve optimal delivery through combination with a suitable delivery scaffold. It is expected to spur innovation in microfluidics through solid cooperation in various fields such as biology, medicine, materials, microfluidics, and biotechnology, going one step further from a simple microfluidic device for drug production. With the advantageous functions presented by microfluidics in mind, the development of smart materials that can be grafted on them is expected to have tremendous potential to accelerate clinical translation by enabling advanced DDSs and patient-specific screening of drugs.

## Acknowledgments

H-J K would like to acknowledge the Basic Science Research Program through the National Research Foundation of Korea (NRF) funded by the Ministry of Education (RS-2023-00240729). This research was supported by the MSIT (Ministry of Science and ICT), Korea under the ITRC (Information Technology Research Center) support program (IITP-2023-RS-2023-00258971) supervised by the IITP (Institute for Information & Communications Technology Planning & Evaluation). This research was also supported by a Korea University Grant (KU S-FRG).

## Data Availability

No new data were created or analysed in this study.
